# Synthesis and Characterization of ZnO Nanowires by Thermal Oxidation of Zn Thin Films at Various Temperatures

**DOI:** 10.3390/molecules17055021

**Published:** 2012-05-02

**Authors:** Mohammad Reza Khanlary, Vahid Vahedi, Ali Reyhani

**Affiliations:** Physics Department of Imam Khomeini International University, Qazvin, 34149-16818, Iran; Email: vahedi_vahid@yahoo.com (V.V.); reyhani@alum.sharif.edu (A.R.)

**Keywords:** zinc oxide, nanowires, thermal oxidation, Zn films, crystal structure

## Abstract

In this research high-quality zinc oxide (ZnO) nanowires have been synthesized by thermal oxidation of metallic Zn thin films. Metallic Zn films with thicknesses of 250 nm have been deposited on a glass substrate by the PVD technique. The deposited zinc thin films were oxidized in air at various temperatures ranging between 450 °C to 650 °C. Surface morphology, structural and optical properties of the ZnO nanowires were examined by scanning electron microscope (SEM), X-ray diffraction (XRD), energy dispersive X-ray (EDX) and photoluminescence (PL) measurements. XRD analysis demonstrated that the ZnO nanowires has a wurtzite structure with orientation of (002), and the nanowires prepared at 600 °C has a better crystalline quality than samples prepared at other temperatures. SEM results indicate that by increasing the oxidation temperature, the dimensions of the ZnO nanowires increase. The optimum temperature for synthesizing high density, ZnO nanowires was determined to be 600 °C. EDX results revealed that only Zn and O are present in the samples, indicating a pure ZnO composition. The PL spectra of as-synthesized nanowires exhibited a strong UV emission and a relatively weak green emission.

## 1. Introduction

Zinc oxide, a wide direct band gap (3.37 eV) semiconductor with a large exciton binding energy (60 meV), has received much attention due to its potential applications in the optoelectronic field [1,2,3]. One-dimensional ZnO nanostructures such as nanowires have been extensively studied for other applications including chemical sensors [4], solar cells [5], blue and ultraviolet (UV) light-emitting diodes [6], transparent electrodes [7] and hydrogen storage [8]. Many techniques have been successfully used to synthesize ZnO nanowires, including sol-gel [9], pulsed laser deposition (PLD) [10], thermal evaporation [11], chemical vapor deposition (CVD) [12], *etc*. Another method to prepare ZnO nanowires, which is more or less used, is thermal oxidation of metallic Zn thin films. Cho *et al*. [13] reported the production of high quality ZnO films by thermal oxidation of metallic Zn. Pure and qualified ZnO films have been prepared by thermal oxidation of metallic Zinc films in air [14,15]. Moreover ZnO nanowires with a mean diameter of 40 nm were synthesized by directly heating Zn powder in an appropriate oxygen atmosphere [16]. Similarly, Tae-Won Kim *et al*. have grown ZnO nanowires with an average diameter of 20 nm by thermal oxidation of predeposited-hexagonal Zn nanoplates on a CaF_2_ (111) substrate [17]. Sirvatsa *et al*. reported the effect of oxygen flow on the growth of vertically aligned ZnO nanorods on Si (100) and sapphire (0001) substrates by using thermal evaporation of pure Zn powder [18]. Dai *et al*. also reported the formation of large-scale ZnO nanowires by the thermal evaporation of metallic zinc powder in the presence of water at a high temperature of about 1,000 °C [19]. However, little work has been reported on the growth of ZnO nanowires on glass substrates by thermal oxidation of Zn thin films at lower temperatures regimes (<600 °C).

In this paper, a very simple, cost effective, non-catalytic growth method for the synthesis of ZnO nanowires is presented. The ZnO nanowires were grown on glass substrates by the thermal oxidation in air of metallic zinc thin films at various temperatures (between 450 and 650 °C). In addition, the growth mechanism of the nanowires is also discussed using the self-catalyzed VLS technique.

## 2. Results and Discussion

**Figure 1:**
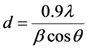
XRD patterns of the ZnO nanowires prepared by the oxidation of Zn thin films at temperatures ranging from 450 °C to 650 °C.

X-ray diffraction (XRD) was employed to investigate the crystal structure of the ZnO nanowires. Figure 1 shows the XRD patterns of the samples prepared by the oxidation of Zn thin films at temperatures between 450 and 650 °C. After the Zn thin films were oxidized at 450 °C and 500 °C, they underwent a partial transformation from Zn to ZnO. The peak at 2θ = 34.56° is related to the ZnO (002). Two peaks at 2θ = 36.53° and 2θ = 77.43° have been found in the XRD pattern which are related to the Zn and assigned as Zn (002) and Zn (004), respectively. The Zn peaks show that the Zn films have a hexagonal close packed crystal structure. 

By increasing the oxidation temperature to 550 °C and higher, metallic Zn is completely oxidized into ZnO. All the diffraction peaks in the patterns can be easily assigned to a ZnO hexagonal wurtzite structure with calculated lattice parameters of a = 3.20 Å and c = 5.19 Å, which are in agreement with the reported standard values (JCPDS No. 01-089-0510). No peaks from impurities, such as Zn, are detected in the patterns, indicating the product is of high purity. The sharp diffraction peak at around 2θ = 34.5° corresponds to the reflection on (002) plane of ZnO and indicate that in the respective films, ZnO nanowires preponderantly with their c-axis orientated. Diffraction peaks for the 600 °C oxidized samples become sharper and the full width at half maximum of the (002) peak is now narrower than others, indicating the excellent crystal quality resulting from this heating process. 

The crystallite sizes of the prepared ZnO nanowires were calculated using the full width at half maximum (FWHM) of the (002) peak employing Scherrer’s formula [20]: 


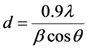
(1)


where 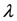, 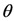 and 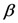 are the X-ray wavelength (0.154 nm), Bragg diffraction angle and FWHM, respectively. The mean grain sizes of the samples were 60 nm, 63 nm, 70 nm for the samples oxidized at 500 °C, 550 °C and 600 °C, respectively, which indicates that the particle size increases with the increasing oxidation temperature. However, by further increasing the temperature to 650 °C, this value decreased, as the mean grain size for the sample oxidized at 650 °C was 60 nm. We think that the results are probably due to the deformation of the glass substrates at 650 °C.

**Figure 2:**
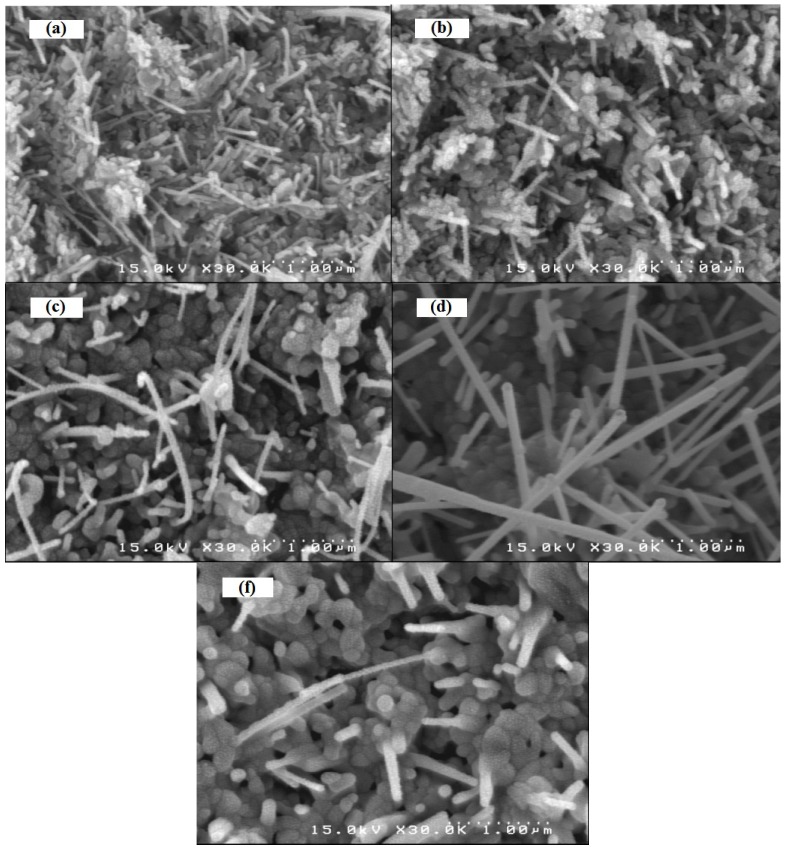
SEM images of the ZnO nanowires prepared by the oxidation of Zn thin films in air for 1 h at temperatures of (**a**) 450 °C, (**b**) 500 °C, (**c**) 550 °C, (**d**) 600 °C, (**f**) 650 °C.

Figure 2 shows the SEM images of the ZnO nanowires prepared by oxidation of the Zn films for 1 h at different temperatures of 450, 500, 550, 600 and 650 °C. Figure 2a shows the ZnO nanowires formed at 450 °C with a diameter of about 38 nm and an average length of 280 nm. By increasing the oxidation temperature, the nanowires produced become longer. Average diameters (obtained by the Microstructure Measurement software) of the nanowires fabricated at 500 °C and 550 °C (Figure 2b,c) are 52 nm and 72 nm and their lengths are 420 nm and 770 nm, respectively. By increasing the oxidation temperature to 600 °C, the nanowire concentration becomes higher, as shown in Figure 2d, with a measured diameter of approximately 87 nm, and a length of approximately 1.4 μm. These data are more accurate than those accused from Deby Scherrer’s formula which is usually just estimation from the sizes. By further increasing the temperature to 650 °C (Figure 2f), length and the concentration of the nanowires were dramatically decreased.

**Figure 3:**
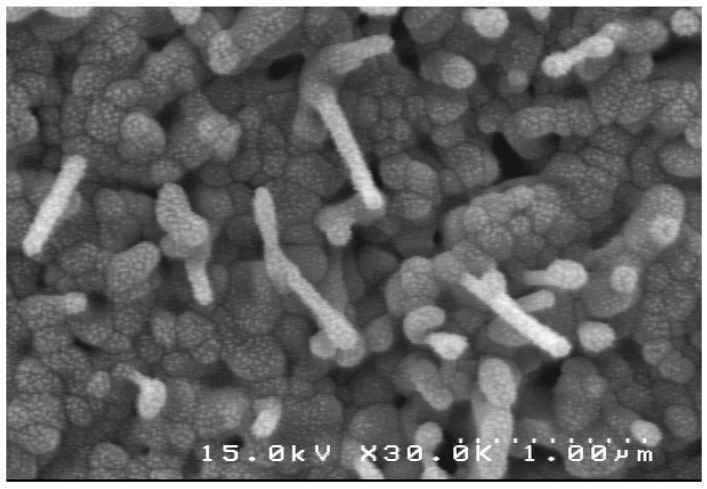
SEM image for showing nanowires growing from individual Zn grains (thin film was oxidized at 600 °C for 30 min in air).

The growth of the 1D nanoforms is generally governed by the catalytic vapor-liquid-solid (VLS) or noncatalytic vapor solid approach. Among the two widely accepted mechanisms for the growth of 1D nanostructures, VLS growth is a catalyst-assisted process, in which a metal catalyst particle acts as a liquid-forming agent [21]. Well-aligned ZnO nanowires arrays have also been synthesized by catalyst-free thermal evaporation methods [22,23]. The vapor-solid (VS) mechanism is generally considered to be responsible for the catalyst-free growth of nanowires [24]. It is also proposed that the catalyst-free growth of ZnO nanowires might include a self-catalyzed vapor-liquid-solid (VLS) process with Zn or ZnO_x_ liquid droplets as catalysts [25]. However, the catalyst-free growth process has not been well understood until now. In the present work, we have prepared ZnO nanowires without any metal catalyst, therefore the growth mechanism of the nanowires can be explained using the self-catalyzed VLS technique. This mechanism consists of two stages: nucleation and growth [26]. When the oxidation temperature of Zinc thin films was ramped higher than the melting point of metallic Zn (melting point of Zn = 419.5 °C), the Zn metal was melted and aggregated to form nano-sized Zn droplets on the surface of the glass substrate. This liquid Zn droplet served as catalyst particles, which were the favorable sites for the absorption of O_2_. The liquid Zn reacts with oxygen and form nanosized ZnO nuclei on the surface of the droplets via a simple chemical reaction 2Zn (l) + O_2_ 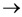 2ZnO. These ZnO nuclei individually further grow in the upper direction in the form of nanowires. From the SEM image (Figure 3), we can observe that the individual Zn grains are oxidized at 600 °C, during a short time of oxidation (~30 min) that shows nanowires growing from individual Zn grains. This growth mechanism ensures that the nanowires are grown with low defects.

**Figure 4:**
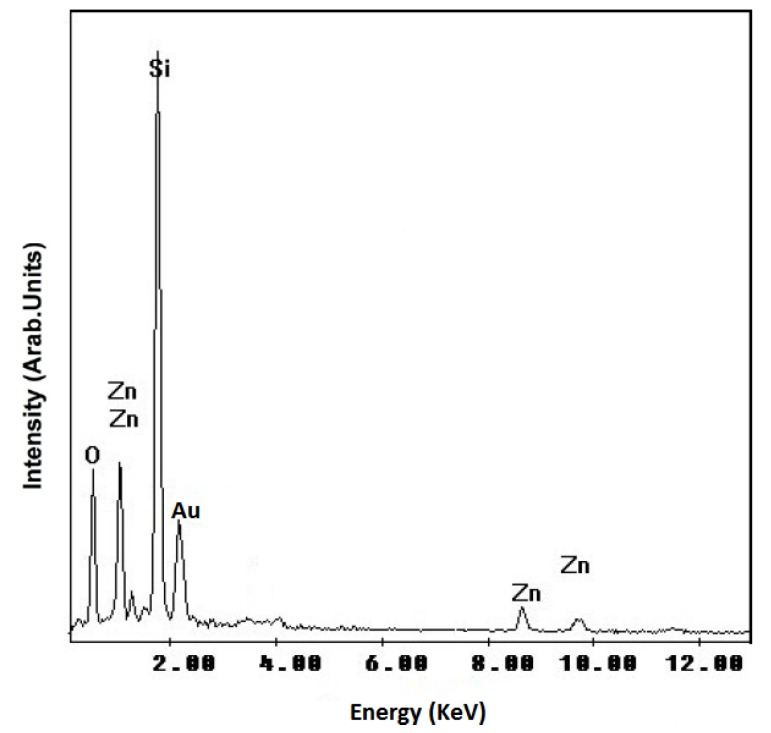
EDX spectrum measured for ZnO nanowires prepared by the oxidation of Zn thin films at a temperature of 600 °C.

An energy dispersive X-ray (EDX) spectrum of the ZnO nanowires is shown in Figure 4. Some features of Zn and O atoms can only be observed in this spectrum. The appearance of Si peak in the spectrum is due to the substrate. Au peaks are from the gold coating. Detecting just Zn and O atoms confirms a high purity of the prepared ZnO, although we cannot deny the possible presence of some minor SiO_2_impurities which were not detectable in the XRD plot.

**Figure 5:**
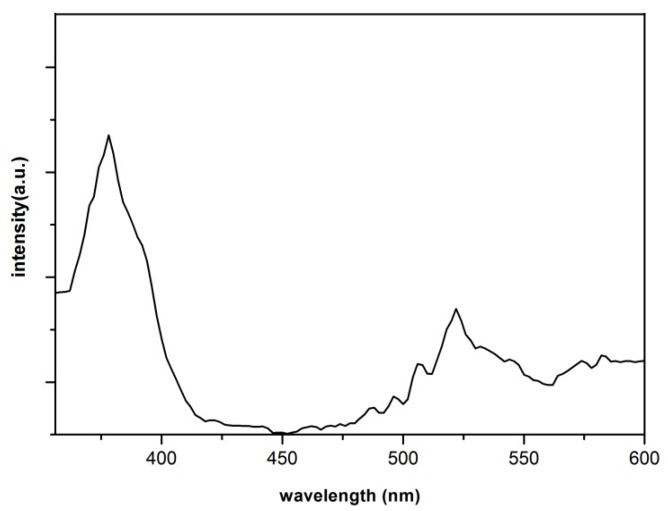
Room temperature’s PL spectrum of the ZnO nanowires prepared by the oxidation of Zn thin films at 600 °C.

To study the optical properties of ZnO nanowires, a room temperature photoluminescence (PL) spectrum of a sample prepared at 600 °C (as an example of the prepared samples) was taken by a Xe light (325 nm) as the excitation source. The spectrum (Figure 5) is composed of an ultraviolet (UV) emission centered at about 377 nm and a broad green emission centered at about 522 nm. The UV emission band can be explained by the near band-edge transition of the wide band gap ZnO nanowires, the recombination of free excitons through an exciton-exciton collision process [27], whereas the peak at 522 nm is due to the deep-level emission (DLE) related to the defects such as oxygen vacancies and Zn interstitials [28,29]. It has been suggested that the DLE corresponds to the singly ionized oxygen vacancy in ZnO and results from the recombination of a photo-generated hole with the singly ionized charge state of this defect [30]. Strong UV emission and relatively weak green emission from the ZnO nanowires confirm that the grown nanowires posses good optical properties with less structural defects and impurities.

## 3. Experimental

Thin films of metallic zinc were deposited by thermal evaporation under vacuum on glass substrates. The source material was zinc metal granulate (Sigma-Aldrich, St. Louis, MO, USA) with a purity of 99.99%. Pressure of the growth chamber was on the order of 10^−5^ Torr. The substrate was kept at room temperature (RT) during the coating process. The distance between the zinc source and the substrate was 20 cm. The Zn films were prepared with some different thickness of 80, 125, 250 and 500 nm. To synthesize ZnO nanowires, Zn films were thermally oxidized in a conventional tube furnace at a temperature of 450–600 °C in air for 1 h or less. The crystal structures of the samples were investigated using X-ray diffraction (XRD, Philips Pw 1800, Eindhoven, The Netherlands) technique with Cuk_α_ radiation (*λ* = 0.154 nm) in the 2θ range of 20°–80°. The surface morphologies and components of the oxidized Zn films were characterized using a scanning electron microscope (SEM, Hitachi S-4160, Tokyo, Japan) and energy dispersive X-ray (EDX) spectroscope (coupled with the Hitachi S-4160), respectively. The photoluminescence (PL, fluorescence spectrometer, Varian Cary Eclipse, Victoria, Australia) spectra of ZnO nanowires were taken at RT using xenon light with a wavelength of 325 nm as an excitation source.

## 4. Conclusions

High quality zinc oxide nanowires were successfully synthesized on glass substrates by thermal oxidation in air of metallic Zn thin films at various temperatures between 450 °C and 650 °C. Actually by annealing at 550 °C and higher temperatures, all of the Zn atoms were transformed to ZnO. XRD analysis demonstrated that the ZnO nanowires has a wurtzite structure with orientation of (002), and the nanowires prepared at 600 °C has better crystalline quality than samples prepared at other temperatures. SEM results indicated that by increasing the oxidation temperature, the dimension of the ZnO nanowires increases. The optimum temperature for synthesizing high density ZnO nanowires was determined to be 600 °C. The growth mechanism of the ZnO nanowires seems more likely to be explained by a self-catalytic VLS process. Room temperature PL spectra of the ZnO nanowires showed a strong UV emission peak located at around 377 nm and a relatively weak green emission at around 522 nm, confirming that the as-grown nanowires possess good optical properties.
